# The Diagnostic Value of Cerebrospinal Fluid Lactate for Detection of Sepsis in Community-Acquired Bacterial Meningitis

**DOI:** 10.3390/diagnostics13071313

**Published:** 2023-03-31

**Authors:** Louisa Nitsch, Stefan Felix Ehrentraut, Marcus Grobe-Einsler, Felix J. Bode, Mohammed Banat, Matthias Schneider, Felix Lehmann, Julian Zimmermann, Johannes Weller

**Affiliations:** 1 Department of Neurology, University Hospital Bonn, 53127 Bonn, Germany; 2 Department of Anesthesiology, University Hospital Bonn, 53127 Bonn, Germany; 3 Department of Neurosurgery, University Hospital Bonn, 53127 Bonn, Germany

**Keywords:** bacterial meningitis, sepsis, cerebrospinal fluid, lactate, outcome

## Abstract

Community-acquired bacterial meningitis conveys significant morbidity and mortality due to intracranial and systemic complications, and sepsis is a major contributor to the latter. While cerebrospinal fluid (CSF) analysis is essential in the diagnosis of bacterial meningitis, its predictive utility for detection of sepsis is unknown. We investigated the diagnostic performance of CSF parameters for sepsis defined by the Sepsis-3 criteria in a retrospective cohort of patients with community-acquired bacterial meningitis. Among 103 patients, 69.5% developed sepsis. CSF lactate was associated with sepsis with an odds ratio of 1.11 (*p* = 0.022), while CSF cell counts, glucose and protein levels were not (all *p* > 0.4). Employing the optimal cutoff of 8.2 mmol/L, elevated CSF lactate resulted in a sensitivity of 81.5% and specificity of 61.5% for sepsis. In exploratory analyses, CSF lactate was also associated with in-hospital mortality with an odds ratio of 1.21 (*p* = 0.011). Elevated CSF lactate might contribute to early diagnosis of sepsis as well as prognostication in patients with community-acquired bacterial meningitis.

## 1. Introduction

Community-acquired bacterial meningitis, an inflammation of the meninges affecting the pia as well as the arachnoid and subarachnoid space, is a disease with significant morbidity and mortality despite widely established diagnostic and therapeutic procedures [1]. It is the most common bacterial infection of the central nervous system with global significance, with the largest burden of meningitis seen in children and in low- or middle-income countries [2]. There is an estimated number of 318,000 deaths and loss of 21.9 million disability-adjusted life years from meningitis per year worldwide, and survivors frequently sustain neurological sequelae [3,4].

In adult patients, the most prevalent causative pathogens are *S. pneumoniae*, *N. meningitidis* and *L. monocytogenes*, which are detected in 72%, 11% and 5% of patients with community-acquired bacterial meningitis, respectively [5]. Other pathogens, such as *S. aureus* and coagulase-negative *staphylococci*, are more frequently encountered following invasive procedures. Patients with bacterial meningitis often have an acute or subacute symptom onset [5]. The clinical ‘classic triad’, consisting of fever, neck stiffness and altered mental status, is present in approximately half of patients only [6]. This poses a diagnostic dilemma, which occurs more frequently in younger compared to older patients [7]. Moreover, differential diagnoses are broad, including other infections of the central nervous system, autoimmune and neoplastic diseases, intracranial hemorrhage or—depending on the geographical setting—malaria [8]. Early recognition and diagnosis is of utmost importance, as a delayed administration of anti-infectious agents is associated with an increased mortality [9]. Therefore, if bacterial meningitis is suspected, further diagnostic procedures need to be initiated immediately, and if this is impossible or only associated with inacceptable delay, empirical anti-infectious treatment should be initiated [10].

Cerebrospinal fluid (CSF) analysis is the mainstay for establishing the diagnosis of bacterial meningitis, which cannot be proven without CSF examination [10]. Standard CSF analysis comprises cell count and differentiation, glucose, lactate and protein concentration, Gram stain and bacterial culture. Characteristic parameters of bacterial infection include low CSF glucose, elevated protein concentration and an increased white blood cell count with usually >80% of neutrophils [5,11,12]. Although CSF parameters vary widely in patients with meningitis, the following individual predictors were shown to detect bacterial meningitis with a high certainty above 99%: a CSF glucose level of less than 40 mg/dL, a CSF-blood glucose ratio of less than 0.23, a CSF protein level greater than 2200 mg/dL, more than 2000/μL leukocytes, or more than 1180/μL neutrophils [13]. In addition, CSF lactate concentration is elevated in bacterial meningitis and has proven to be efficient in differentiating between viral and bacterial meningitis [14,15,16], but it is less accurate in patients with ventriculostomies or for differentiating patients with other central nervous system diseases, such as herpes encephalitis or seizures [10,17]. Other markers, such as CSF ferritin and albumin index, also seem to be useful in differentiating bacterial and viral meningitis [18]. Detection of the causative pathogen is also possible with CSF Gram stain or CSF culture in up to 85% of patients, but anti-infectious pretreatment reduces the diagnostic yield [5,12,19]. Furthermore, polymerase chain reaction is widely available as a routine diagnostic in meningitis patients and allows for a rapid detection of the pathogens.

If the diagnosis of bacterial meningitis is established or treatment warrants initiation before a definitive diagnosis can be made, empiric anti-infectious treatment according to published guidelines and the local antibiotic resistance situation should be administered immediately [10]. Following detection of the causative agent, the choice of antibiotic agents must be adjusted accordingly. In addition, early treatment with dexamethasone improves the outcome in high-income countries in acute bacterial meningitis due to *S. pneumoniae* or other bacteria, with the exception of *L. monocytogenes* [8,20].

Even in presence of adequate and timely treatment, infection and bacteremia can lead to sepsis, a frequent complication in bacterial meningitis and other infectious diseases with a high risk of multiple organ dysfunction and death. The Third International Consensus Definitions for Sepsis and Septic Shock (Sepsis-3) define sepsis as life-threating organ dysfunction caused by a dysregulated host response to infections [21]. The early detection of septic patients is important to decrease morbidity and mortality. A widely used score score to identify patients with sepsis is the Sequential (Sepsis-related) Organ Failure Assessment (SOFA). The SOFA score is based on six different sub-scores, including the respiratory, cardiovascular, hepatic, coagulation, renal and neurological system each scored from 0 to 4, with higher scores reflecting worsening of organ dysfunction [22]. An increase in the SOFA score of 2 points or more represents a clinical operationalization of organ dysfunction and allows for the diagnosis of sepsis in the setting of bacterial infection, which was associated with an in-hospital mortality of more than 10% [21].

Despite being available in virtually all patients with confirmed bacterial meningitis, CSF parameters have not been evaluated as a predictive parameter for presence of sepsis in bacterial meningitis. We here aim to analyze the diagnostic utility of standard CSF parameters for the diagnosis of sepsis as defined by Sepsis-3 and show that elevated CSF lactate was predictive of sepsis in our cohort of adult patients with community-acquired bacterial meningitis.

## 2. Materials and Methods

### 2.1. Study Design and Patient Characteristics

We retrospectively included all consecutive adult patients (age ≥ 18 years) treated at our tertiary university medical center between January 2009 and October 2022 according to the following criteria: diagnosis of community-acquired bacterial meningitis, based on cerebrospinal fluid (CSF) examination with either a proven pathogen or with CSF abnormalities highly suggestive for bacterial meningitis (CSF-blood glucose ratio < 0.23, glucose level < 40 mg/dL, CSF protein level > 2200 mg/dL, leukocytes > 2000/μL, polymorphonuclear leukocytes > 1180/μL) [13,23]. Community-acquired meningitis was assumed in absence of a hospital stay and invasive spinal or cerebral procedures 4 weeks before diagnosis.

Following lumbar puncture, the CSF was immediately analyzed as part of routine diagnostics using standard methods. CSF glucose content was determined with the hexokinase method, protein content was analyzed with the turbidimetric method, lactate was measured using enzymatic colorimetric assays based on the oxidation of lactate to pyruvate and white blood cell counts were evaluated using flow cytometry. 

Clinical information, including age, sex, symptoms at admission, infectious focus and agent, immunocompromised state, length of hospital stay and discharge condition, were registered. An immunocompromised state was defined as a history of alcoholism, diabetes mellitus, splenectomy, HIV infection or immunosuppressive medication. Functional outcome on discharge was rated according to the modified Rankin Scale (mRS) and further dichotomized as follows: mortality, mRS 6; favorable outcome, mRS 0 to 2. 

Sepsis due to community-acquired bacterial meningitis was defined as an increase of the SOFA score of 2 or higher upon admission or until 48 h later [21]. The SOFA score was assessed as described previously [22]. If PaO_2_ was not available, the PaO_2_/FiO_2_ ratio was substituted by the SpO_2_/FiO_2_ ratio as described previously [24].

The study was reviewed and approved by the Ethics Committee of the Rheinische Friedrich-Wilhelms University Bonn, Bonn, Germany (no. 164/21). Written informed consent for participation was waived in accordance with national legislation and institutional requirements. 

### 2.2. Statistical Analysis

Standard descriptive statistics were used for all data presented in the manuscript and parameters were compared between groups by student’s *t*-test, Mann–Whitney U test, Fisher’s exact test and chi square test, where appropriate. Univariable logistic regression models were calculated to analyze the utility of CSF parameters for detection of sepsis. Results are presented as odds ratios (OR) with 95% CI. Receiver operating characteristic (ROC) curves were constructed for significant predictors. The area under the curve was determined and the corresponding 95% confidence intervals were calculated as defined by DeLong. Optimal cutoff values were determined with Youden’s J index. The significance level was set to alpha = 0.05 and all analyses were two-sided. Statistical analyses were carried out with R (R Core Team, 2022, version 4.2.1).

## 3. Results

### 3.1. Patient Characteristics and Clinical Outcome

A total of 103 consecutive patients with community-acquired bacterial meningitis treated at the authors’ institution between January 2009 and October 2022 were included in the analysis. The median age was 60 years (interquartile range [IQR], 51–72) and 47.6% were female. Upon admission, headache was reported in 66.6%, fever in 67.4% and neck stiffness in 58.9%, while an altered consciousness was noted in 64.4% of patients. At least one of these four cardinal symptoms was present in 95.1% and all four were noted in 15.5%. The most frequent focus of infection was mastoiditis, otitis or sinusitis in 32.0%. *S. pneumoniae* was the most common pathogen in 42.6% of patients, while no pathogen was identified in 19.4%. An immunosuppressive condition was noted in 29.1% of patients.

CSF analysis revealed abnormalities in all patients. The median CSF lactate was 11.4 mmol/L (IQR, 7.2–15.0), the median CSF glucose was 17.5 mg/dL (IQR, 3.0–47.3), the median protein content was 3284 mg/L (IQR, 1674.0–5775.5) and the median CSF white blood cell count was 2162/μL (350–6297). Among patients where CSF cell differentiation was performed (n = 90), the median CSF lymphocyte count was 203/μL (IQR, 72–454) and the medium absolute CSF neutrophil count was 1279/μL (IQR, 195–5304), while the medium relative CSF neutrophil count was 88% (IQR, 70–95).

All patients were treated with antimicrobial agents and 57.1% received dexamethasone (78.6% of patients with *S. pneumoniae* infection). Sepsis as defined by the Sepsis-3 criteria was present in 69.5% of patients within 48 h. Among these, Sepsis-3 criteria were fulfilled most frequently during the first 24 h (53.7%, later: 15.8%). Patients with sepsis more frequently presented with altered consciousness, while the causative pathogen less frequently remained unknown compared to patients without sepsis (Table 1). The median length of hospital stay was 24 days (IQR, 16–38). The median mRS score on discharge was 2.5 (IQR, 1–5). In-hospital mortality was 14.7% and 50% of patients achieved a favorable outcome, defined as a mRS of 0 to 2. 

### 3.2. Prognostic Utility of CSF Values

CSF analytics for patients with and without a diagnosis of sepsis are presented in Table 2. In patients with sepsis, the CSF lactate was higher and the glucose level lower (Table 2). In logistic regression analysis, elevated CSF lactate was associated with presence of sepsis with an odds ratio of 1.11 (95% confidence interval, 1.02–1.21, *p* = 0.022) per increase in mmol/L, while CSF glucose and protein content as well as CSF cell counts were not (all *p* > 0.4, Table 3).

**Figure 1:**
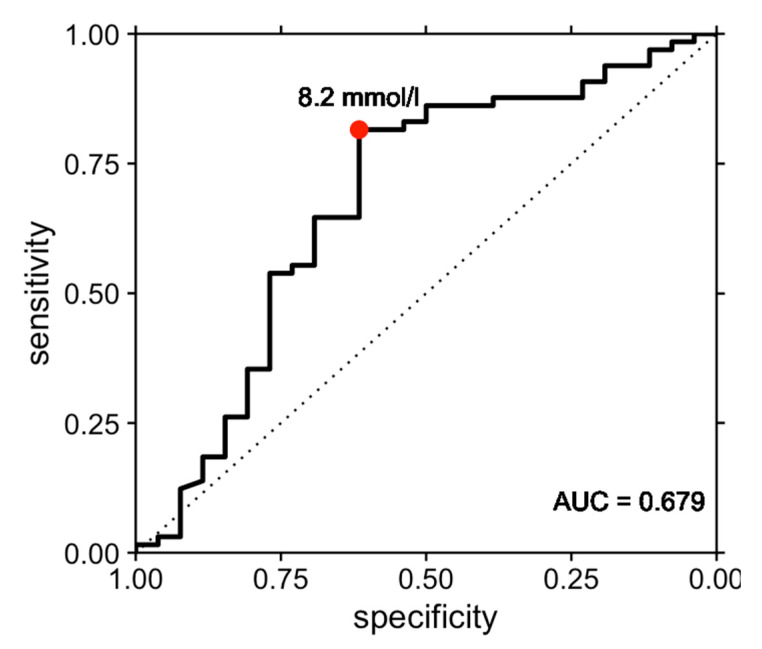
Receiver operating characteristic (ROC) curve illustrating the performance of cerebrospinal fluid lactate in the prediction of sepsis in bacterial meningitis. The ROC curve had an area under the curve (AUC) of 0.679 (95% confidence interval, 0.544–0.814). The optimal threshold value of 8.2 mmol/L as determined by Youden’s J index was associated with a sensitivity of 81.5% and a specificity of 61.5% for prediction of sepsis.

The resulting ROC curve had an area under the curve of 0.679 (95% confidence interval, 0.544–0.814, Figure 1). The optimal threshold value of 8.2 mmol/L as determined by Youden’s J index was associated with a sensitivity of 81.5% and a specificity of 61.5%. The associated positive predictive value of a CSF lactate threshold value of 8.2 mmol/L for sepsis was 84.1%, while the negative predictive value was 57.1%.

To confirm the clinical relevance of these findings, we performed further exploratory analyses constructing logistic regression models, including CSF parameters to predict mortality in our cohort. Here, elevated CSF lactate was associated with in-hospital mortality with an odds ratio of 1.12 (95% confidence interval, 1.03–1.24, *p* = 0.011), while CSF glucose and protein content as well as CSF cell counts were not (all *p* > 0.05, Table 4).

## 4. Discussion

We investigated the diagnostic performance of CSF parameters for sepsis in a retrospective cohort of patients with community-acquired bacterial meningitis. Among 103 consecutive patients, approximately 70% developed sepsis according to the Sepsis-3 criteria, most frequently during the first 24 h. CSF lactate was associated with sepsis, while other standard CSF parameters such as glucose and protein levels as well as cell counts were not. The optimal CSF lactate cutoff of 8.2 mmol/L resulted in a sensitivity of 81.5% and a specificity of 61.5%. In patients with bacterial meningitis, CSF analysis is always performed unless contraindicated and measuring lactate concentration is a widely available, cheap and rapid diagnostic test [10,25]. Accordingly, it represents an easily accessible parameter for the prediction of sepsis in patients with community-acquired bacterial meningitis. To our knowledge, this is the first study to show the importance of CSF parameters in the early detection of sepsis in meningitis, as CSF lactate was associated with sepsis in patients with community-acquired meningitis and might thus aid the identification of most severely affected patients. In further exploratory analyses, CSF lactate was also associated with in-hospital mortality, further underlining the importance of this finding.

Lactate is produced during anaerobic glycolysis, which occurs in most tissues and results in conversion of pyruvate to lactic acid by the enzyme lactate dehydrogenase [26]. Since the 1960s, serum lactate has evolved into a well-established marker of disease severity, particularly among patients with sepsis [27,28]. While serum lactate is tested in the emergency department and intensive care unit on a routine basis, CSF lactate is ordered less frequently. Following its first description in 1917 by Levinson, the importance of CSF lactate for the distinction of bacterial from non-bacterial meningitis is now well established, while it is less accurate for differentiating patients with other central nervous system diseases, such as herpes encephalitis, meningeosis neoplastica or seizures [10,29,30].

Of note, elevated CSF lactate levels result from anaerobic central nervous system glycolysis and are independent of serum lactate levels, as the charged ion crosses the blood-brain barrier only very slowly [26,31]. CSF lactate levels in bacterial meningitis are high, typically comparable to those in empyema [32]. Potential sources of elevated CSF lactate levels in bacterial meningitis include direct bacterial production, cerebral edema, inflammation and cerebral ischemia [26]. Animal experiments point towards the brain parenchyma rather than the subarachnoid space as the source of elevated CSF lactate [33]. Leukocytes do not seem to contribute significantly to increased CSF lactate, as leukocyte depletion did not result in decreased CSF lactate levels in experimental pneumococcal meningitis [34]. Taken together, these results suggest bacterial production and tissue hypoxia as the most probable sources of lactate production. Following this argumentation, CSF lactate is expected to increase with severity of infection, explaining the association with sepsis in bacterial meningitis in our study. Accordingly, CSF lactate has been shown previously to correlate with poorer outcome both in animal models and retrospective patient cohorts, which was also shown in our cohort [35,36,37]. In addition to being a mere marker of disease severity, elevated CSF lactate might also directly contribute to it, as *N. meningitidis* and *S. pneumoniae* can use lactate as a carbon source in absence of glucose, thus sustaining bacterial growth as CSF glucose levels fall rapidly [32].

Although CSF lactate was reported to correlate with protein content and CSF cell counts to some extent in an etiologically heterogeneous cohort, the prognostic impact of other standard CSF parameters is much less pronounced [38]. Lower CSF cell counts are associated with poorer outcome, which might reflect an inadequate host response to the infection, while CSF protein content and the blood glucose ratio were not associated with outcome in large retrospective studies [5,12]. 

Sepsis, a frequent complication in bacterial meningitis, conveys significant morbidity and mortality as well as an impaired quality of life among survivors [39,40]. Accordingly, clinical signs of systemic infection, such as tachycardia, hypotension, increased C-reactive protein and reduced platelet count, were associated with poor outcome in retrospective cohorts with community-acquired bacterial meningitis [5,12]. Timely antibiotic treatment is of course mandatory in bacterial meningitis, irrespective of sepsis diagnosis [10]. Prediction of sepsis is nevertheless crucial to guide early and adequate supportive treatment, including fluid resuscitation and hemodynamic monitoring [41]. In addition, more complex early sepsis-directed therapy to adjust cardiac preload, afterload and contractility to balance oxygen delivery with oxygen demand is essential in patients with severe sepsis and septic shock, and early goal-directed therapy was shown to convey significant short-term and long-term benefits [42]. This might be especially pronounced in older patients, as age represents a risk factor for both sepsis and bacterial meningitis [43,44,45]. Intriguingly, cardiorespiratory failure was more frequently observed as the cause of death in older patients, while younger patients more frequently died from intracranial complications [7]. The results of the study may help to rapidly identify patients with sepsis due to community-acquired meningitis and allocate adequate treatment resources.

In our study, about 70% of patients with community-acquired bacterial meningitis developed sepsis according to the Sepsis-3 criteria. Following the publication of these criteria, we could not identify any other studies reporting the incidence of sepsis among patients with bacterial meningitis. A study from 2006 by the Dutch Meningitis Cohort reported an incidence of sepsis or respiratory failure of 30–50% in pneumococcal meningitis, but without using internationally accepted criteria due to lack of data [46]. The same group reported that hypotension as a surrogate parameter for sepsis was associated with lower CSF white blood cell counts (low: 44% vs. high: 11%) in meningococcal meningitis, but not in pneumococcal meningitis [12]. Apart from these reports, there is a surprising paucity of data on sepsis in adult patients with bacterial meningitis, and a lack of predictors of sepsis in this setting. 

Outside from bacterial meningitis, more than 250 biomarkers have been evaluated over the last few decades, and while C-reactive protein and procalcitonin as host-response biomarkers are frequently used in clinical care, none of them precisely differentiates between sepsis and sepsis-like syndromes [47]. C-reactive protein is an acute phase protein synthesized in the liver upon activation of the immune system with widespread clinical use, but while its increase is sensitive for bacterial infections and meningitis, it is by far not specific [48]. Serum procalcitonin has a remarkably quick dynamic, rising 3-4 h after an inflammatory stimulus [49]. It is of established importance in the early diagnosis of sepsis in general with sensitivity of up to 80% and specificity of up to 75% [50]. However, while increased procalcitonin levels are strongly associated with bacterial infection and sepsis, it also fails to reliably differentiate sepsis from other non-infectious causes of systemic inflammatory response syndrome in critically ill adult patients, and there is no defined procalcitonin cutoff value for diagnosis of sepsis [47,51]. Of note, it might be useful for the differential diagnosis of meningitis [52,53]: serum procalcitonin was reported to distinguish between bacterial and viral meningitis with a specificity of up to 100% [54,55]. CSF procalcitonin analysis is a promising new diagnostic marker in bacterial meningitis, which might prove especially helpful in patients with a reduced diagnostic yield from standard CSF investigations, for example, after neurosurgical procedures or following antibiotic pretreatment [56,57]. Future work should also investigate novel CSF parameters, such as procalcitonin or ferritin, for prediction of sepsis in patients with bacterial meningitis.

The present study has all limitations of a retrospective single-center analysis. It was performed at a German tertiary academic medical center, which limits generalization, and statistical power is reduced by the sample size. Further, SOFA criteria were evaluated retrospectively. As the diagnostic utility of CSF lactate for prediction of sepsis was evaluated in the context of community-acquired bacterial meningitis, our data do not allow extrapolation of the findings to other diseases associated with elevated CSF lactate. 

## 5. Conclusions

Approximately 70% of patients with community-acquired bacterial meningitis developed sepsis according to the Sepsis-3 criteria. Elevated CSF lactate levels upon diagnosis were associated with sepsis and higher mortality in these patients. To our knowledge, this is the first study to show the importance of CSF lactate in the early detection of sepsis in meningitis. Prospective analyses are warranted to confirm these findings and refine the reported cut-off values.

## Figures and Tables

**Table 1:** Baseline characteristics of patients with community-acquired bacterial meningitis with and without sepsis as defined by the Sepsis-3 criteria. *p* values for comparisons between patients with and without sepsis are presented.

	No Sepsis	Sepsis	*p* Value
Median Age, years	56 (48–66) ^1^	61 (52–71) ^1^	0.09
Female Sex, %	62.1	40.9	0.08
Baseline symptoms, %			
Headache	79.3	59.3	0.09
Fever	67.9	64.4	0.81
Neck stiffness	69.0	50.0	0.11
Altered consciousness	31.0	78.1	<0.001
Infectious focus, %			
Mastoiditis, otitis, sinusitis	37.9	33.3	0.82
Endocarditis	0	10.6	0.19
Pneumonia	0	7.6	0.32
Causative pathogen, %			
*S. pneumoniae*	31.0	53.0	0.07
*S. aureus*	6.9	3.0	0.58
*L. monocytogenes*	6.9	3.0	0.58
*E. coli*	0	4.5	0.55
Unknown	34.5	13.6	0.027
Immunosuppressive condition, %	24.1	28.8	0.80

^1^ Interquartile range.

**Table 2:** Median cerebrospinal fluid (CSF) parameters and interquartile range in patients with community-acquired bacterial meningitis with and without sepsis.

CSF Parameter	No Sepsis	Sepsis	*p* Value
Lactate, mmol/L	6.9 (5.0–12.4)	13.0 (9.2–16.3)	0.008
Glucose, mg/dL	23 (12–52)	16 (2–45)	0.33
Protein, mg/L	1731 (799–5299)	3576 (2118–5400)	0.04
White blood cell count, /μL	2387 (279–4325)	2167 (417–6378)	0.62
Lymphocyte count, /μL	229.5 (77.75–456)	260 (72–497)	0.79
Granulocyte count, /μL	1654 (223–3700)	1357 (192–6060)	0.67
Relative granulocyte count, %	85.2 (75.4–92.1)	88.0 (68.9–94.6)	0.88

**Table 3:** Association of cerebrospinal fluid (CSF) parameters with sepsis in univariable logistic regression analysis. CSF lactate was significantly associated with sepsis with an odds ratio (OR) of 1.11 per mmol/L increment. For all other CSF parameters presented here, there was no significant association with sepsis detected.

CSF Parameter	Unit	Odds Ratio	95% CI ^1^	*p* Value
Lactate	Per mmol/L	1.11	1.02–1.21	0.022
Glucose	Per mg/dL	0.99	0.98–1.01	0.43
Protein	Per mg/L	1.00	0.92–1.11	0.94
White blood cell count	Per 1000/μL	1.02	0.99–1.11	0.57
Lymphocyte count	Per 1000/μL	1.48	0.89–4.92	0.41
Granulocyte count	Per 1000/μL	1.02	0.99–1.14	0.57
Relative granulocyte count	Per %	0.92	0.13–5.58	0.93

^1^ Confidence interval.

**Table 4:** Association of cerebrospinal fluid (CSF) parameters with mortality in univariable logistic regression analysis. CSF lactate was significantly associated with mortality with an odds ratio (OR) of 1.21 per mmol/L increment. For all other CSF parameters presented here, there was no significant association with mortality detected.

CSF Parameter	Unit	Odds Ratio	95% CI ^1^	*p* Value
Lactate	Per mmol/L	1.21	1.03–1.24	0.011
Glucose	Per mg/dL	0.98	0.96–1.01	0.20
Protein	Per mg/L	1.04	0.95–1.12	0.37
White cell blood count	Per 1000/μL	1.05	1.00–1.16	0.32
Lymphocyte count	Per 1000/μL	1.81	1.14–3.70	0.052
Granulocyte count	Per 1000/μL	1.00	0.99–1.02	0.52
Relative granulocyte count	Per %	0.28	0.04–2.18	0.21

^1^ Confidence interval.

## Data Availability

The anonymized data included in the manuscript are available from the corresponding author upon reasonable request and according to local data protection and privacy laws.

## Abbreviations

AUC	area under the curve
CI	confidence interval
CSF	cerebrospinal fluid
*E. coli*	*Eschierichia coli*
FiO_2_	fraction of inspired oxygen
*H. influenzae*	*Haemophilus influenzae*
IQR	interquartile range
*L. monocytogenes*	*Listeria monocytogenes*
mRS	modified Rankin Scale
*N. meningitidis*	*Neisseria meningitidis*
OR	odds ratio
PaO_2_	partial pressure of oxygen in the arterial blood
*S. aureus*	*Staphylococcus aureus*
*S. pneumoniae*	*Streptococcus pneumoniae*
SOFA	Sequential Organ Failure Assessment
SpO_2_	oxygen saturation
